# Supplementary material for: North Atlantic warming over six decades drives decreases in krill abundance with no associated range shift
Source: Commun Biol. 2021 May 31;4:644. doi: 10.1038/s42003-021-02159-1 (PMC8166933; doi:10.1038/s42003-021-02159-1)
Supplement: Supplementary file 2 — Supplementary Information [file 42003_2021_2159_MOESM2_ESM.pdf]

Supplementary Information

North Atlantic warming over six decades drives decreases in krill abundance with no associated range shift

Martin Edwards[1*,] Pierre Hélaouët[2], Eric Goberville[3], Alistair Lindley[2], Geraint Tarling[4], Michael T. Burrows[5], Angus Atkinson[1]

*Corresponding author: globalplankton@gmail.com

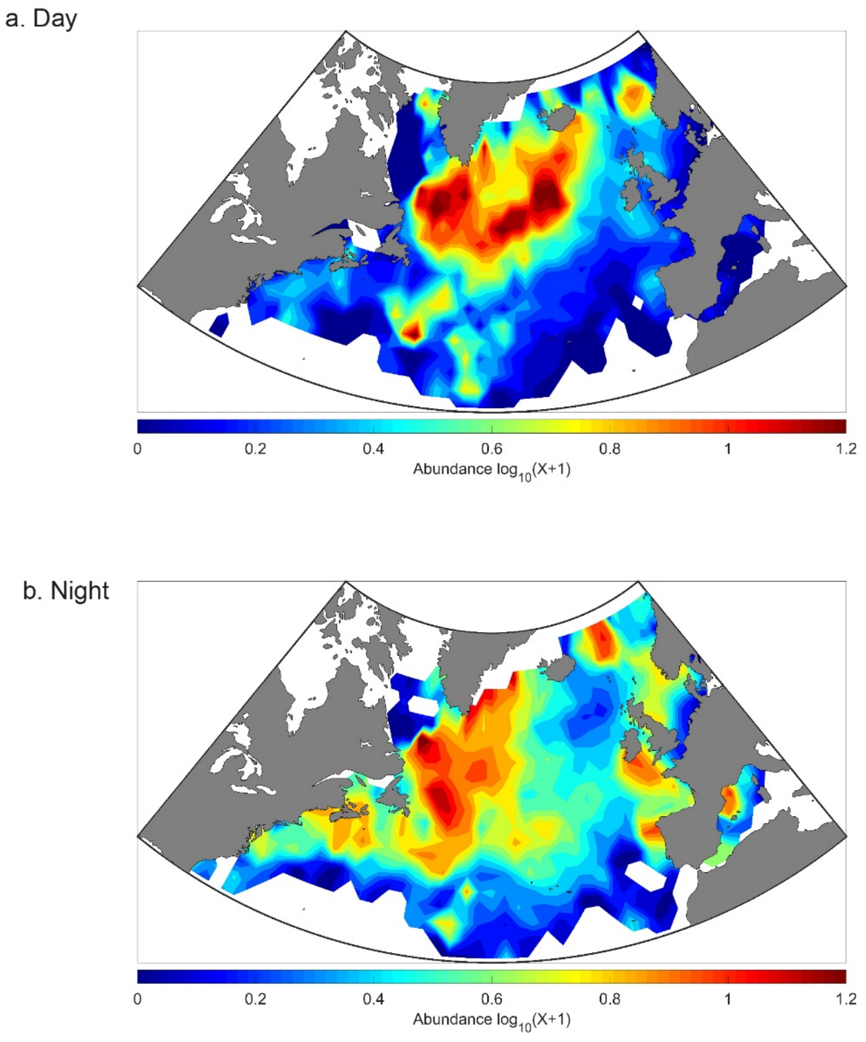

Supplementary figure 1: **Geographical distribution of euphausiids in the North Atlantic between day (a) and night samples (b)**.
