# Supplementary material for: North Atlantic warming over six decades drives decreases in krill abundance with no associated range shift
Source: Commun Biol. 2021 May 31;4:644. doi: 10.1038/s42003-021-02159-1 (PMC8166933; doi:10.1038/s42003-021-02159-1)
Supplement: Supplementary file 3 — Reporting Summary [file 42003_2021_2159_MOESM3_ESM.pdf]

nature research

Corresponding author(s): *Double-blind peer review submissions: write DBPR and your manuscript number here instead of author names.*

Last updated by author(s): *YYYY-MM-DD*

Reporting Summary

Nature Research wishes to improve the reproducibility of the work that we publish. This form provides structure for consistency and transparency in reporting. For further information on Nature Research policies, see our Editorial Policies and the Editorial Policy Checklist.

Statistics

For all statistical analyses, confirm that the following items are present in the figure legend, table legend, main text, or Methods section.

n/a	Confirmed	
[ ]	[x]	The exact sample size (*n*) for each experimental group/condition, given as a discrete number and unit of measurement
[x]	[ ]	A statement on whether measurements were taken from distinct samples or whether the same sample was measured repeatedly
[x]	[ ]	The statistical test(s) used AND whether they are one- or two-sided<br>*Only common tests should be described solely by name; describe more complex techniques in the Methods section.*
[x]	[ ]	A description of all covariates tested
[ ]	[x]	A description of any assumptions or corrections, such as tests of normality and adjustment for multiple comparisons
[ ]	[x]	A full description of the statistical parameters including central tendency (e.g. means) or other basic estimates (e.g. regression coefficient) AND variation (e.g. standard deviation) or associated estimates of uncertainty (e.g. confidence intervals)
[x]	[ ]	For null hypothesis testing, the test statistic (e.g. *F*, *t*, *r*) with confidence intervals, effect sizes, degrees of freedom and *P* value noted<br>*Give P values as exact values whenever suitable.*
[x]	[ ]	For Bayesian analysis, information on the choice of priors and Markov chain Monte Carlo settings
[x]	[ ]	For hierarchical and complex designs, identification of the appropriate level for tests and full reporting of outcomes
[ ]	[x]	Estimates of effect sizes (e.g. Cohen's *d*, Pearson's *r*), indicating how they were calculated

Our web collection on statistics for biologists contains articles on many of the points above.

Software and code

Policy information about availability of computer code

Data collection	Standard MATLAB code were used in the analysis (no custom code used)
Data analysis	Standard MATLAB code were used in the analysis (no custom code used)

For manuscripts utilizing custom algorithms or software that are central to the research but not yet described in published literature, software must be made available to editors and reviewers. We strongly encourage code deposition in a community repository (e.g. GitHub). See the Nature Research guidelines for submitting code & software for further information.

Data

Policy information about availability of data

All manuscripts must include a data availability statement. This statement should provide the following information, where applicable:

- Accession codes, unique identifiers, or web links for publicly available datasets
- A list of figures that have associated raw data
- A description of any restrictions on data availability

The data that support the findings in this study are available from the open data portal. See (https://www.mba.ac.uk/blog/oceans-open-data) for more information.

Field-specific reporting

Ecological, evolutionary & environmental sciences study design

All studies must disclose on these points even when the disclosure is negative.

Study description	Long-term and spatial study of Euphausiids abundancne in the North Atlantic based ion observations
Research sample	258,919 plankton samples were used in the analysis, this contained information on adult Euphausiid collected in the North Atlantic Ocean by the Continuous Plankton Recorder Survey.
Sampling strategy	All data collected by the Continuous Plankton Recorder in the North Atlantic were used in the study (258,919 samples)
Data collection	The Continuous Plankton Recorder (CPR) Survey is a long-term, sub-surface marine plankton monitoring programme consisting of a network of CPR transects towed monthly across the major geographical regions of the North Atlantic
Timing and spatial scale	Data used in the analysis were monthly data collected from 1958 to 2017. The spatial scale ranged from the meso to macro scale.
Data exclusions	No data was excluded from the analysis
Reproducibility	Not applicable at this scale of observational study
Randomization	Not relevant to this observational style study
Blinding	Not applicable at this scale of observational study

Did the study involve field work? ☐ Yes ☒ No

Reporting for specific materials, systems and methods

We require information from authors about some types of materials, experimental systems and methods used in many studies. Here, indicate whether each material, system or method listed is relevant to your study. If you are not sure if a list item applies to your research, read the appropriate section before selecting a response.

Materials & experimental systems

n/a	Involved in the study
☒	☐ Antibodies
☒	☐ Eukaryotic cell lines
☒	☐ Palaeontology and archaeology
☒	☐ Animals and other organisms
☒	☐ Human research participants
☒	☐ Clinical data
☒	☐ Dual use research of concern

Methods

n/a	Involved in the study
☒	☐ ChIP-seq
☒	☐ Flow cytometry
☒	☐ MRI-based neuroimaging
